# Childhood Leukemia in Small Geographical Areas and Proximity to Industrial Sources of Air Pollutants in Three Colombian Cities

**DOI:** 10.3390/ijerph17217925

**Published:** 2020-10-29

**Authors:** Laura Andrea Rodriguez-Villamizar, Feisar Enrique Moreno-Corzo, Ana María Valbuena-Garcia, Claudia Janeth Uribe Pérez, Mary Ruth Brome Bohórquez, Héctor Iván García García, Luis Eduardo Bravo, Rafael Gustavo Ortiz Martínez, Jürg Niederbacher Velásquez, Alvaro R. Osornio-Vargas

**Affiliations:** 1 Department of Public Health, Universidad Industrial de Santander, Bucaramanga 680002, Colombia; avalbuena@cuentadealtocosto.org; 2 Public Health Observatory of Santander, Fundación Oftalmológica de Santander, Floridablanca 681003, Colombia; rasief@gmail.com (F.E.M.-C.); rafael.ortiz@foscal.com.co (R.G.O.M.); 3 Cuenta de Alto Costo, Fondo Colombiano de Enfermedades de Alto Costo, Bogotá 110111, Colombia; 4 Population Based Cancer Registry of the Metropolitan Area of Bucaramanga, Universidad Autónoma de Bucaramanga, Bucaramanga 681003, Colombia; curibep@unab.edu.co; 5 Population Based Cancer Registry of Antioquia, Gobernación de Antioquia, Medellín 050015, Colombia; mary.brome@antioquia.gov.co; 6 Cancer Institute Las Américas, Medellín 050022, Colombia; hgarcia@une.net.co; 7 Population Based Cancer Registry of Cali, Universidad del Valle, Cali 760043, Colombia; luis.bravo@correounivalle.edu.co; 8 Department of Pediatrics, Universidad Industrial de Santander, Bucaramanga 68002, Colombia; jurgnied@uis.edu.co; 9 Department of Pediatrics, University of Alberta, Edmonton, AB T6G 1C9, Canada; osornio@ualberta.ca

**Keywords:** leukemia, childhood, cluster analysis, air pollution, industrial pollution, Colombia

## Abstract

Acute leukemia is the most common childhood cancer and has been associated with exposure to environmental carcinogens. This study aimed to identify clusters of acute childhood leukemia (ACL) cases and analyze their relationship with proximity to industrial sources of air pollution in three capital cities in Colombia during 2000–2015. Incident ACL cases were obtained from the population cancer registries for the cities of Bucaramanga, Cali, and Medellín. The inventory of industrial sources of emissions to the air was obtained from the regional environmental authorities and industrial conglomerates were identified. The Kulldorf’s circular scan test was used to detect city clusters and to identify clusters around industrial conglomerates. Multivariable spatial modeling assessed the effect of distance and direction from the industrial conglomerates controlling for socioeconomic status. We identified industrials sectors within a buffer of 1 km around industrial conglomerates related to the ACL clusters. Incidence rates showed geographical heterogeneity with low spatial autocorrelation within cities. The spatio-temporal tests identified one cluster in each city. The industries located within 1 km around the ACL clusters identified in the three cities represent different sectors. Exposure to air pollution from industrial sources might be contributing to the incidence of ACL cases in urban settings in Colombia.

## 1. Introduction

Leukemia is the most common childhood cancer worldwide [1]. According to the most recent report of the Global Cancer Observatory, it is estimated that 7745 new cases of leukemia were diagnosed in 2018 in Latin America and the Caribbean in children under 15 years old. The age-standardized rate of leukemia in this region is estimated in 49 cases per million, only exceeded by North America and Europe (58 and 50 cases per million, respectively). In terms of mortality due to childhood leukemia, the Latin America and the Caribbean region has an estimated age-standardized mortality rate of 20 deaths per million children under 15 years old, the highest mortality rate for leukemia shared with the Asia region [2].

In Colombia it is estimated that acute childhood leukemia (ACL) accounts for 36% of the total incident childhood cancer cases, followed by the central nervous system tumors (16%) and lymphomas (14%) [3]. The estimated age-standardized incidence rate for leukemia in Colombia was 68.4 cases per million for children between 0 and 14 years old during 1992–2013 [4]. According to the long-term data of the population-based cancer registry of Cali, Colombia, the incidence of childhood leukemia had an annual percent change of 1.0 (95% confidence interval (CI): 0.2–1.8) between 1977 and 2011, while the mortality had an annual percent change of –1.2 (95% CI: −2.6–0.3) during the same time [5].

The causes of ACL and their biological pathways are not yet well understood. However, there is evidence that genetic conditions, infectious and environmental exposures are the most important contributors for leukemia [6,7]. Environmental factors associated with leukemia incidence include ionizing radiation, pesticides exposure, parental smoking, air pollution, and household chemicals [8]. The report of the World Health Organization on air pollution and child health highlights the fact that air pollution has a terrible impact on child health and survival as 93% of children live in environments with air pollution levels above the WHO guidelines [9].

Several studies conducted in Europe and the United States have evaluated the relationship between air pollution exposure and the childhood cancer risk, especially of ACL. The first published study (1989) was conducted in Denver, USA, and found an increased risk of childhood cancer and leukemia using traffic counts at the home address at the time of diagnosis as pollution exposure [10]. Most of studies in the following 15 years focused mainly on assessing traffic-related air pollution using case-control and ecologic designs and provided mainly negative evidence for the relationship with childhood cancer [11]. Then, a meta-analysis of seven case-control studies assessing specifically residential traffic exposure and risk of childhood leukemia found positive associations among studies using a postnatal exposure window and no association among studies using prenatal exposure window [12]. Another comprehensive review and meta-analysis of outdoor air pollution and childhood leukemia risk included six ecologic and 20 case-control studies using different exposure measurements. They found an increased risk of childhood leukemia related to high traffic density, increased risk of acute lymphoblastic leukemia related to nitrogen dioxide exposure, and of acute myeloid leukemia related to benzene exposure [13].

In contrast, studies estimating the effect of industrial air pollution on childhood cancer are less common. Initial ecologic studies from Great Britain found a geographic association of leukemia and solid cancers with petroleum-derived volatiles and effluents of combustion engines [14]. Studies published in the last five years in Spain suggest a relationship between industrial pollution and childhood cancer. A population-based case-control study assessing the effect of residential proximity to industrial pollution found an increased risk of childhood leukemia for children living near to specific types of industries [15]. A study assessing proximity to air-polluting industries using cluster analysis and small geographical areas in the Region of Murcia, found a possible association between proximity to specific industries and childhood cancer [16]. More evidence from other world regions is needed before establishing the relationship between industrial pollution and childhood leukemia.

The use of spatial epidemiological studies is useful for exploring geographical patterns of disease, opening the possibility for studying potential associations with underlying conditions that might differ across places. A recent study identified spatial clusters of childhood leukemia in five regions of Colombia when using municipalities as spatial units of analysis. The clusters identified included four of the five largest cities in Colombia: Bogotá, Cali, Medellín, and Bucaramanga [17]. However, little is known about the clustering of cases within cities and their relation with industrial pollution sources. This study aimed to identify clusters of ACL cases, using a spatial analysis based on small geographical areas, and analyze their relationship with proximity to industrial sources of air pollution in three capital cities in Colombia during 2000–2015.

## 2. Materials and Methods 

### 2.1. Study Areas, Population, and Geographical Data

This ecologic and spatial analysis study centers in Colombia, a country located at the northern extreme of South America, with a population of approximately 48 million people. The study areas included the cities of Bucaramanga, Cali, and Medellín. These three cities were chosen because they were previously identified as municipalities with clusters of leukemia [17] and the existence of population-based cancer registries. Bucaramanga, the capital city of the department of Santander locates in the northeast of Colombia and has a population of approximately 529,000 people. Cali, the capital city of the department of Valle del Cauca, locates in the southwest of the country and has a population of approximately 1,823,000 people. Medellín, the capital city of the department of Antioquia, locates in the northwest of the country and has approximately 2,373,000 people [18].

Data for the population at risk came from the National Census 2005, National Department of Statistics (DANE, for its name in Spanish). Census population annual projections of children less than 15 years were obtained from DANE for the study period (2000–2015) for the three cities [19]. Population data was available from Census 2005 at section, sector, and block level. The census sector (CS) is the intermediate geographical territorial unit for an urban area that corresponds to the area delimited by the census perimeter and is made up of census blocks, which is the smaller geographical census unit. Census sectors do not have a specific size or population. We used CS as the spatial unit for cluster analysis in this ecologic study as it offers an adequate small-area unit of analysis for health outcomes and preserves the privacy of cases. We performed linear interpolation for estimating the annual population for each CS during the study period based on the linear interpolation of DANE projections of the population between 0 and 14 years old for the cities.

We worked with the geographic coordinates (latitude and longitude) of population-based CS centroids. The distance and direction between the location of each "industrial conglomerate" and the CS centroids were calculated using the Euclidean distance (in meters) and angle (in geodetic degrees) in ArcGIS 10.6.1^®^ (Environmental Systems Research Institute ESRI, Redlands, CA, USA). The maps of the three cities at CS level were obtained from the DANE Geoportal public website [20], and the spatial data were created in ArcGIS 10.6.1^®^ using the projection of Colombia in mode Custom Azimuth Equidistant and Datum WGS 1984. The socioeconomic status data were obtained from the municipalities at the neighborhood level and then the predominant socioeconomic status (SES) from the neighborhoods in each CS (usually between 2 and 3), represented the whole CS SES. The SES data uses the DANE “socioeconomic strata” classification of the socioeconomic resources of residential places [21], which range from one to six being one the strata with higher socioeconomic deprivation.

### 2.2. Acute Childhood Leukemia Data

In Colombia there are four Population-Based Cancer Registries validated by and reporting to the International Agency for Research on Cancer (IARC). These registries are located in the cities of Cali, Bucaramanga, Manizales, and Pasto, and offer high-quality information about cancer for these cities. The population-based cancer registry of the metropolitan area of Bucaramanga was created in 2000 and registers cases of Bucaramanga and the other three municipalities of the metropolitan area. The cancer registry of Cali was created in 1962, is the oldest cancer registry in Latin America and the pioneer in implementing cancer registry methods in Colombia [22]. The cancer registry of Antioquia is a population-based cancer registry created in 2000, which register cancer cases for Medellín and all municipalities in Antioquia.

The ACL data were obtained from the population-based cancer registries of the Bucaramanga metropolitan area, Cali and Antioquia, for the cities of Bucaramanga, Cali, and Medellín, respectively. Incident confirmed ACL cases diagnosed from 1 January 2000, to 31 December 2015, from residents in the cities were included in the study. For the cancer registries and this study, resident ACL cases are those who have been living six months or more in the city before the date of ACL diagnosis. The information available in the cancer registries for ACL cases that were used for this study included the type of ACL, sex, date of birth, date of diagnosis, and place of residence at the time of diagnosis. When the address or neighborhood of residence was not available in the cancer registries files, the information was obtained by the cancer registry professionals from the health institutions who reported the cases. The address or neighborhood of residence at the time of diagnosis served to determine the CS of residence at the time of diagnosis for ACL cases.

### 2.3. Industrial Facilities Data

The list (inventory) of industrial sources of emissions to the air was obtained from the regional environmental authorities for the cities: Corporación para la Defensa de la Meseta de Bucaramanga (CDMB) in Bucaramanga, Corporación Autónoma Regional del Valle del Cauca (CVC) and Departamento Administrativo de Gestion del Medio Ambiente (DAGMA) in Cali, and Area Metropolitana del Valle de Aburrá (AMVA) in Medellín. There were 32 industrial facilities identified in Bucaramanga, 289 in Cali, and 144 in Medellín. Data obtained included the address, coordinates, and industrial activity type of industrial facilities. We used distance to facilities as proxy measure of exposure because quantitative data of emissions to the air were not available for the industries during the study time window (2000–2015). Historical data for industrial emissions is available only for one year (2011) for Cali and two years (2011 and 2013) for Medellin. The industrial facilities were aggregated according to their geographical location in 4, 26 and 14 “industrial conglomerates” within Bucaramanga, Cali, and Medellín, respectively. The coordinates of the centroids (latitude and longitude) of the “industrial conglomerates” areas were used as the industrial location for focal cluster and multivariable spatial analysis of the cases.

### 2.4. Statistical Analysis

Descriptive analysis was conducted by calculating mean annual specific incidence rates of ACL and age-sex standardized rates by city. The standardized rates calculation used the direct method using the Colombian population with intervals of 5 years of age up to 14 years as the standard population. Bayesian smoothed incidence morbidity ratios were also calculated by CS to reduce heterogeneity when estimating the risk of ACL and then mapped as choropleth maps. We used the global Moran’s Index for calculating global spatial autocorrelation and local Moran’s Index for identifying spatial clusters of CS with high ACL rates. Statistical analyses were calculated in Stata 15^®^ and maps were created using ArcGIS 10.6.1^®^.

We used the Kulldorff’s circular scan test to detect local clusters within the cities and its focal mode for identifying clusters around “industrial conglomerates” [23]. We ran the Kulldorff’s test using a retrospective space-time analysis, scanning for clusters with high rates using a discrete Poisson model. The spatial unit was the CS of residence, and the time unit was the year of diagnosis. We set as the upper limit for the size of those corresponding to a circle that included the 25% of the total number of ACL cases. Then centroid coordinates of the “industrial conglomerates” created grids for the focused tests. The significant level for the test was 0.05. This hypothesis test for clustering was selected based on its good performance to detect compact clusters of rare diseases and its widespread use in spatial epidemiological studies [24]. We used the SaTScan ^®^ software version 9.6 (Kulldorff M. and Information Management Services, Inc., Boston, MA, USA).

We also conducted multivariable modeling with spatial variables using the CS as the unit of analysis aimed to assess the effect of distance and direction from the “industrial conglomerate” on the ACL incidence. The distance variable represented the measured distance in Km from the centroid of the “industrial conglomerate” to the centroid of each CS. The direction was modeled using the sine (longitude) and cosine (latitude) functions of the angle between both centroids. We used a Poisson model using the log of the expected cases by CS as an offset variable. A basic model was built for the effect of distance and subsequent models added other spatial functions (direction and interactions between distance and direction). Then, the effects of spatial variables were adjusted by the predominant SES of the CS. We used the Akaike information criterion as parameters for model selection. Models were built separately for all the “industrial conglomerates” that showed significant clusters in the focused Kulldorf’s scan tests. These analyses were conducted using Stata 15^®^ (Stata Corporation, College Station, TX, USA).

We identified the industrial sectors in proximity to the ACL clusters by identifying the industrial facilities located within a buffer of 1 km around the centroid of the “industrial conglomerates” that showed statistical significance of the distance variable in the multivariable model and were included within the spatio-temporal clusters identified for each city.

### 2.5. Research Ethics

All procedures performed in this study followed the national and international ethical standards and the Ethical Committee for Research of the Universidad Industrial de Santander (CEINCI-UIS) granted ethical approval of the study. Informed consent was not required due to the nature of the study and data sources. For those cancer cases missing place of residence information, data were obtained by the cancer registries from the administrative health databases of the health provider institutions. The cancer registries managed all personal data and the information was anonymized and compiled to a small area level (census sector) for analysis.

## 3. Results

**Figure 1:**
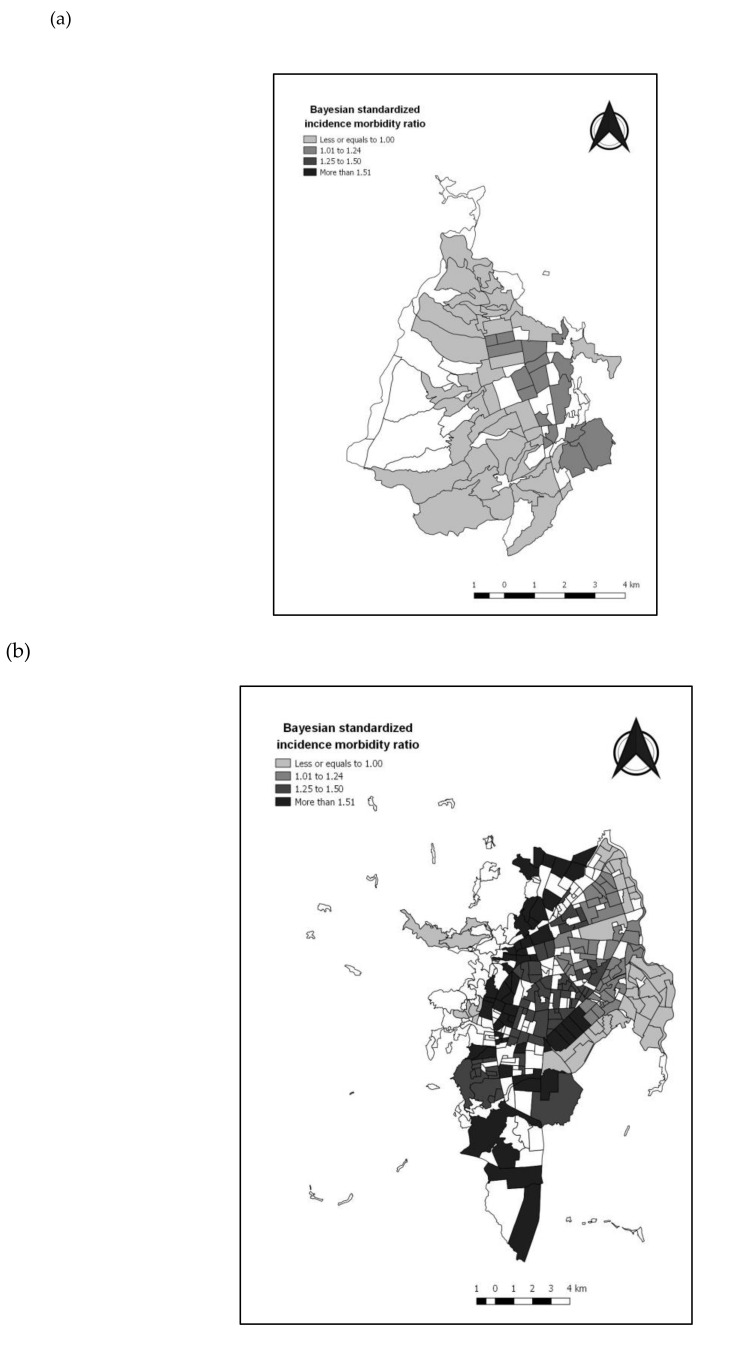

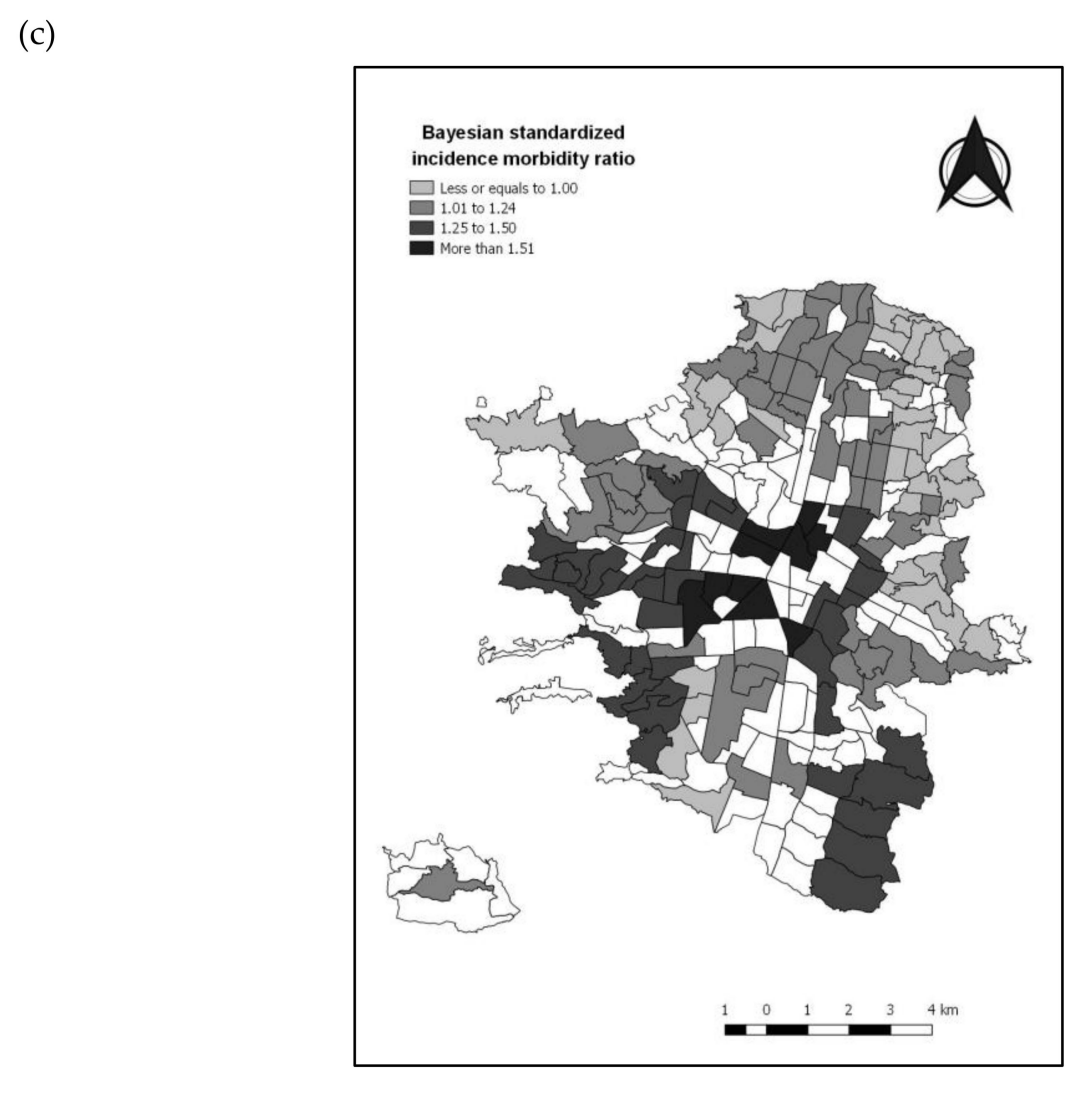
Bayesian smoothed rates of acute childhood leukemia in urban sectors of three capital cities, Colombia 2000–2015. (**a**) Bucaramanga; (**b**) Cali; (**c**) Medellín.

There were 140,469 and 314 confirmed ACL cases during 2000–2015 in Bucaramanga, Cali, and Medellin, respectively. The annual mean age-standardized incidence rate per million children under 15 years was higher in Bucaramanga (70.03), followed by Cali (52.68) and Medellín (41.24). Most cases occurred in males with a mean age of six years old at the time of diagnosis. Table 1 shows the characteristics of ACL cases in terms of age, sex, time of diagnosis, and the number of census sectors (CS) with ACL cases. Bayesian smoothed rates of ACL by CS shows geographical heterogeneity within cities (Figure 1). The Moran’s Index was –0.005 (*p* = 0.349) for Bucaramanga, 0.038 (*p* < 0.001) for Cali, and 0.041 (*p* < 0.001) for Medellín, suggesting a low spatial autocorrelation of ACL cases among CS in Cali and Medellín.

**Figure 2:**
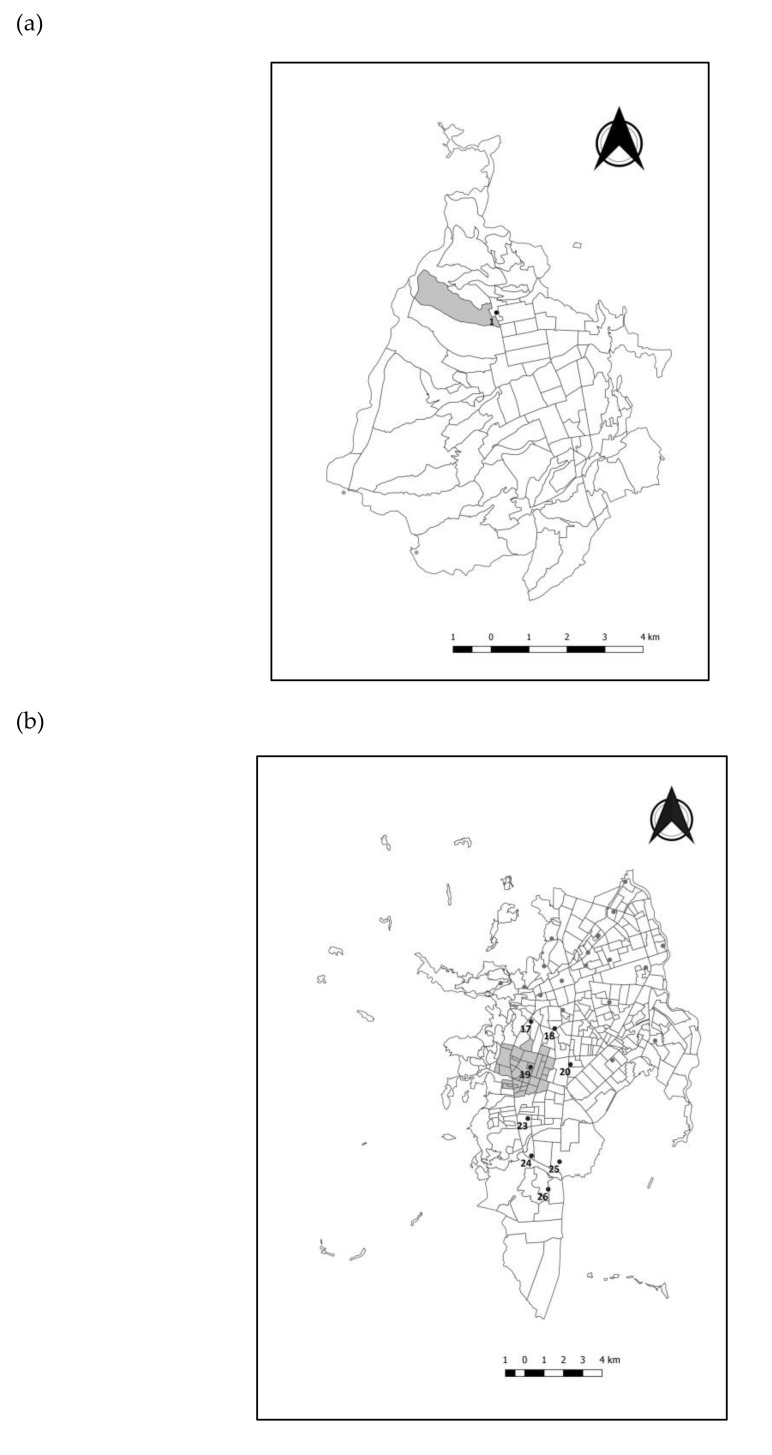

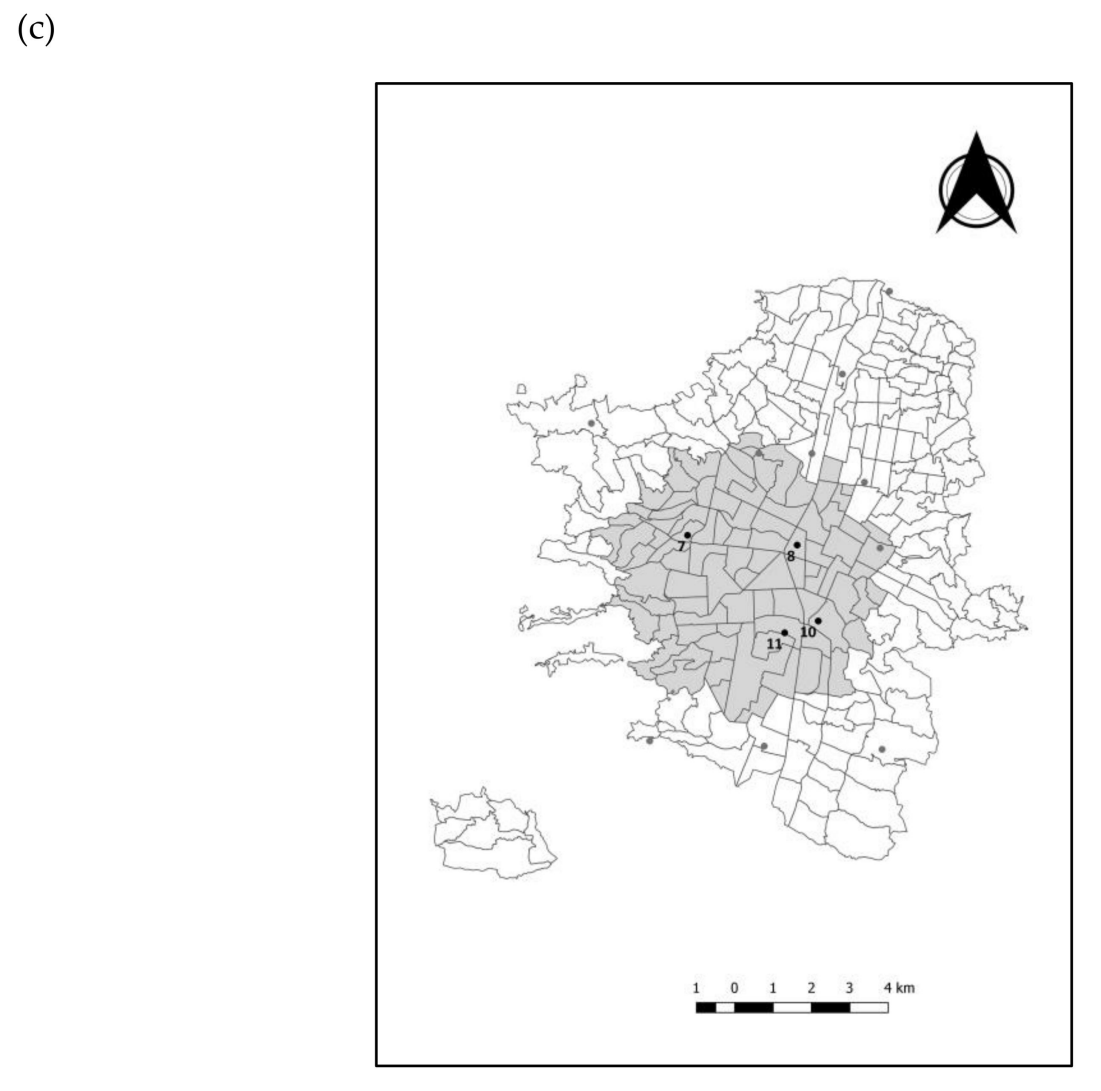
Localized spatio-temporal clusters of acute childhood leukemia and industrial conglomerates in urban sectors of three capital cities, Colombia 2000–2015. (**a**) Bucaramanga; (**b**) Cali; (**c**) Medellín. Note: industrial conglomerates are shown as points and the conglomerate’s numbers are presented only for conglomerates with statistically significant result in cluster analysis.

Table 2 shows a summary of Kulldorff’s circular scan tests results. The test for localized spatial clusters identified one in Bucaramanga, two in Cali and Medellín. The spatio-temporal tests identified one cluster in Bucaramanga (for the period 2010–2011), one in Cali (for the period 2003–2006), and one in Medellin (for the period 2002–2005). Figure 2 illustrates the location of clusters and their geographical relation with locations of “industrial conglomerates” within cities. Supplementary tables (Tables S1–S3) present details of the clusters identified in the cities. The ACL spatio-temporal cluster identified in Bucaramanga locates in the northwest of the city; in Medellín the cluster is located in the city center and extends towards the southwest; and in Cali the cluster is identified at west of the city (Figure 2). The results of the local Moran’s Index also identified the same spatial cluster in the west of Cali. In Medellin, the cluster was identified in the center of the city, which is a more localized region included in the spatial cluster identified by the circular scan test immediately above the industrial conglomerate 8. An additional CS with a high rate was identified at south of the city in the south border of the spatial cluster identified by the scan test.

Results of multivariable modeling with spatial variables using the CS as the unit of analysis are in Table 3. Controlling for spatial direction (latitude and longitude of the angle between the industrial conglomerate and the CS centroid) and predominant SES in the CS, there were industrial conglomerates spatially related to a higher incidence of ACL: one in Bucaramanga, eight in Cali, and four in Medellín. The industrial facilities within a buffer of 1 Km of the Bucaramanga’s industrial conglomerate #1 are related to the processing of raw agricultural products. The industrial facilities within a buffer of 1 Km of the Cali’s industrial conglomerates #17–20 include predominantly energy power plants fueled with diesel (mainly in conglomerate #19 located within the cluster 1) with some paints and food processing industries. The Cali’s industrial conglomerates #23–26 are not included within any ACL spatio-temporal cluster. In Medellín the industrial conglomerate #7 includes facilities of the chemical and food sector; conglomerate #8 includes facilities of the metalworking, pharmaceutical, and textile sector; conglomerate #10 includes facilities of the textile and car repairing sector, both using tinctures and paints; and conglomerate #11 includes facilities of the textile, rubbers, and leather sector. According to the inventory of industrial sources of emissions to the air of the cities, the industrial conglomerates spatially related to the clusters were stable across the time window of the study (2000–2015) as most of them were in place at the beginning of the 2000 decade and were still active in the inventory list for 2012.

## 4. Discussion

This study assessed the presence of space and space-time clustering of ACL cases in three capital cities of Colombia and their proximity to industrial air pollution sources. Using census sectors as the small-geographical area units for the spatial analysis, we identified one ACL cluster in each city; we also identified the predominant industrial sectors in proximity to these clusters. The lack of data of estimated industrial emissions to the air in the three cities during most of the study period leads to uncertainty in the results. Therefore they should be considered as preliminary evidence. To the best of our knowledge, this is the first study assessing the relationship between ACL clusters and proximity to industrial facilities at a small geographical level in South America.

Industrial sectors associated with ACL clusters seem to be different across the three cities. In Cali, the main ACL cluster was located in the proximity of an industrial conglomerate with the predominance of energy power plants. These energy-generating plants are fueled by diesel and besides potential benzene emissions probably generate a relevant electromagnetic field. Some ecological, case-control and cohort studies conducted in Asia and Europe have found associations between residential proximity to electromagnetic fields of high voltage power plants and lines with the increased risk of childhood leukemia [25]. In Medellin, there is a large cluster of ACL cases in proximity to industrial conglomerates that are located within the city around the river with the predominance of metalworking, chemical, and textile-leather industries with the use of tinctures and paints. Emissions of these industries might include volatile organic compounds, such as benzene, that have been implicated in leukemia incidence [26]. In Bucaramanga, the least industrialized of the three cities, the cluster identified was small and in proximity to an industrial conglomerate that processes raw agricultural products; during this process volatile organic compounds might be produced and pesticides from raw products might be emitted to the air. Studies conducted in Europe and North America have found associations between increased risk of leukemia and brain tumors and exposure to agricultural and domestic pesticides [25,27].

Some childhood cancers have shown variations in incidence according to the extent of socio-economic development. In the case of leukemia is has been observed that incidence increases with countries’ socioeconomic development [28]. The placement and expansion of industries might explain this situation that in the early phases of the development are not well controlled by government authorities. Therefore, territories start growing by mixing industrial and residential areas. Populations around industrial areas are predominantly poor, which places them at double risk for childhood cancer: poverty and exposure to industrial activities and emissions [29]. In this regard, it is essential to clarify that for the cities included in the study, and in general for Colombia, the national and local government during the last decade have been working in the delimitation and separation of industrial areas as a public policy to regulate industries and protect public health. Our results suggest that the 2000–2015 ACL incidence could be reflecting previous exposures that were stable and still present at the beginning of the 2010s decade. The urban growth of most capital cities in Colombia was not planned, and despite land use definitions, control for avoiding and eliminating industrial sources from residential places is relatively recent. Thus, industries were located in some cities within residential areas, or industrial areas were surrounded by residential new developments related to city growth. Thus, people living in cities might have been exposed to industrial air pollution emission for long periods.

Spatial clusters of leukemia have been identified in other countries. The EUROCLUS project assessed the spatial clustering of childhood leukemia in 17 European countries between 1980 and 1989 and found evidence of clustering within small census areas with intermediate population density [30]. In Spain, using a case-control study and place of residence at birth in five autonomous regions between 1996 and 2011 there was no evidence of the clustering of childhood cancer [31]. In contrast, clustering of acute childhood leukemia at the place of residence during pregnancy was identified in the region of Murcia, Spain between 1998 and 2013, suggesting that environmental exposure in utero might be important determinants for ACL [32]. In Switzerland a nationwide study assessed the presence of clustering childhood cancers using both, place of residence at birth and at the time of diagnosis during 1985–2010, and identified significant space-time clusters of childhood leukemia at birth but no at diagnosis, suggesting an etiologic factor present in early life [33]. Some studies in the United States have identified clusters of leukemia in Ohio [34] and Nevada [35] where the spatio-temporal patterns identified at the time of diagnosis suggest the hypothesis of a possible infectious cause. In California a case-control cluster analysis identified evidence of clustering of acute lymphoblastic leukemia diagnosed at 2–6 years of age between 1997 and 2007 in the San Francisco Bay Area by using the birthplace of children [36].

In Latin America, there is also some evidence of clustering for childhood leukemia: in Argentina, significant clusters of childhood leukemia were identified in the province of Cordoba using the residential address at the time of diagnosis between 2004 and 2013 [37]; in Mexico, clustering of ACL at the time of diagnosis between 2010 and 2014 was identified in the city of Guadalajara [38]; in Colombia a nationwide study at municipality level and time of diagnosis identified ACL clusters in five regions [17]. A recent systematic review and pooled analysis of space-time clustering studies of childhood cancer included 47 studies of childhood leukemia published before July 2016 and concluded that significant clusters are present at both time of diagnosis and birth; the clusters were identified especially for children aged 0–5 years for a spatial lag of 5 km and temporal lag of 6 months, suggesting that the pattern of clustering close to the time of diagnosis might be compatible with an infection cause to be identified [39].

Childhood leukemia has been associated with proximity to industrial complexes in other countries. During the 1990s Knox reported in England a childhood leukemia cluster close to railways, petrochemical plants and steelwork industries [40]. Clusters of childhood leukemia and solid cancers occurred in proximity to oil refineries and petroleum-related facilities, motor car factories and repair facilities, industries that use kilns and furnaces, including industries that produced smoke, gases and effluents from internal combustion engines; the identified clusters manifested when considering the place of birth between 1953 and 1980 [14]. In Taiwan, Weng et al., identified an increased risk of childhood deaths for leukemia between 1995 and 2005 in municipalities with the highest levels of petrochemical air pollution [41]. In Spain, a study found an increase of risk in childhood leukemia in children living up to 2.5 km of industries related to glass and mineral fibers, surface treatment using organic solvents, galvanization, and production and processing of metals. The study was a population-based case control study of childhood leukemia was conducted in four autonomous regions for the period 1990–2011 using place at the time of diagnosis and assessing the effect of residential proximity to both industrial and urban pollution, and taking into account industrial groups and substances released. [15].

A review of 25 studies of childhood cancer and residential proximity to potential environmental hazards concluded that for leukemia the environmental exposures with evidence of association were: (1) traffic-related pollution, petrochemical plants, gas stations or car repair garages, in relation with benzene exposure; (2) pesticides exposures; (3) nuclear power plants; (4) and landfill sites [42]. A meta-analysis of exposure to benzene in utero and early life exposure identified evidence of consistent associations of childhood leukemia with different metrics of benzene exposure, including traffic-pollution, occupational and household use of solvents [43]. Our results for the city of Medellín are consistent with these studies as industries around the cluster identified might be related to benzene emissions. The finding of one cluster in the city of Cali related to energy power plants fueled by diesel might reflect exposure to benzene and electromagnetic fields (EMFs). In this regard, different studies have addressed the association between residential magnetic fields and childhood leukemia, and a pooled analysis reported small but consistent increased risk with EMFs exposures above 0.3 uT [44]. In the case of the city of Bucaramanga, the cluster identified might be associated with emissions of facilities’ furnaces, exposure to benzene for being an area of high traffic, or indirect exposure to pesticides presents in agricultural raw products. The evidence of an association between exposure to pesticides and childhood leukemia has been specific for outside herbicide exposure and indoor residential insecticides [45]; a recent case-control state-wide study California for ACL cases between 1998–2011 showed evidence of elevated risk of acute lymphocytic leukemia for exposure to a variety of pesticides during pregnancy. However, this evidence is specific for rural and not urban areas [46].

Our findings and those reported in the literature, suggest that the potential associations relate to specific chemicals and physical factors instead of individual sectors. These observations also point out that each city has a different pattern of hazards that may call for regulatory actions tailored to tackle each location’s specific conditions.

There are some strengths worth mentioning of this study. First, we used ACL high-quality data coming from population-based cancer registries with rigorous quality control [22]. The high coverage of the cancer registries along with the high percentage of geolocation of census sectors for the cases allowed us to include in the analysis more than 85% of cases for Bucaramanga and more than 95% of cases for Cali and Medellín. Second, we used spatial analysis tools for descriptive, hypothesis testing, and multivariable modeling that allowed to identified clusters and estimate effects of proximity to industrial conglomerates. Third, we controlled the proximity effect by the potential confounding effect of the socioeconomic status, which is recognized as a critical confounder at the ecological and individual level for studies assessing the effect of environmental exposures on childhood cancers [47].

This study’s main limitation is the lack of quantitative data on air pollutants emissions from the industries during the study period. Unfortunately, there is no historical data available for industrial emissions for the decade 2000–2010 in the three cities included in the study. For the period 2011–2015, there is only one year available (2011) for Cali and two years (2011 and 2013) for Medellin reporting pollutants that might be relevant to our study. According to the total emission’s inventory for Medellin and the Aburrá Valley in 2011, industries emitted 89% of SO_x,_ 20% of PM_2.5_ and 10% of volatile organic compounds (VOC) emissions with higher concentrations in the center and south of the city. The textile, ceramic and glass and food sectors contributed around 80% of the estimated annual industrial emissions of PM_10_, VOC, and SOx. These 2011 emission patterns relate to the location and type of industries found spatially related to Medellin’s ACL clusters in our study [48]. The Cali’s 2011 industrial emissions inventory reports that the estimated highest releases of SOx and PM_10_ belong to industries from the textile sector while metalworking sector released most of the VOC. More than 70% of SOx and PM_10_ emissions occurred in the city center and >70% of VOCs were released north in the city. These patterns have no relation to the ACL cluster locations found in this study [49]. Emissions inventories and characterization of air pollutant composition became available for the cities of interest in recent years. For this reason, our spatial analysis is based only on distances, and therefore it has to be considered an exploratory study with no causal association. In contrast to Europe and North America, most Pollution Release and Transfer Registries are recent or starting to operate in developing countries. Therefore the availability of these data will provide critical information for further studies with enough quantitative data.

Knowing that residential mobility might affect children’s exposure adds another limitation of this study since we lacked ACL cases residential address during pregnancy and at birth. The use of residential location at the time of diagnosis allowed us to identify potential hazards close to the time of diagnosis but not necessarily those related to early life exposures. Although we did not systematically analyze mobility in all cities, a sub-analysis of residential mobility in Bucaramanga [50] showed that most cases were living in the same address in the previous two years, and half of them lived in the same address since birth. Another limitation is the lack of control for the potential confounding effect of the traffic-related air pollution and the residential proximity to gas stations at the small-area level since this information is not available for the cities. Finally, we did not include the wind direction as part of the spatial multivariable models’ spatial variables. Therefore we were not able to assess its effect on the ACL incidence. Further studies incorporating the chemical nature of the industrial emissions, meteorological and traffic-related air pollution variables would support the hypothetical associations described in this paper.

## 5. Conclusions

Acute leukemia is the most common childhood cancer in Colombia, and we identified clusters of ACL cases in three departmental capital cities. These clusters were associated with proximity to specific industrial conglomerates within the cities but with different industrial sectors patterns. Our results suggest that exposure to air pollution from industrial sources might contribute to the incidence of ACL cases in Colombia’s urban settings. These results are based only on proximity (distance) analysis and provide preliminary evidence with no causal association. Further studies incorporating recent available data on pollution emissions to the air and dispersion models would provide more robust evidence. Therefore, this study’s results reinforce the need for a continuous commitment of environmental and health authorities to maintain and improve the instruments needed to measure, surveil, regulate and control industrial emissions to the air to protect the children’s current and future health.

## Figures and Tables

**Table 1:** Characteristics of acute childhood leukemia cases in three capital cities, Colombia 2000–2015.

Variable	Bucaramanga	Cali	Medellín
Estimated mean population (children under 15 years)	126,483	559,217	481,612
Total incident cases	140	469	314
Age in years			
Median (IQ range)	5 (3–10)	6 (3–10)	6 (3–11)
Mean (SD)	6.1 (4.04)	6.45 (4.28)	6.7 (4.30)
Male (%)	84 (60.00)	251 (53.52)	192 (61.15)
Age groups *n* (%)			
0–4 years	60 (42.86)	193 (41.15)	127 (40.45)
5–9 years	44 (31.43)	148 (31.56)	83 (26.43)
10–14 years	36 (25.71)	128 (27.29)	104 (33.12)
Time period cases *n* (%)			
2000–2007	71 (50.71)	243 (51.82)	79 (25.15)
2008–2015	69 (49.29)	226 (48.18)	235 (74.85)
Specific incidence rate (annual mean per million)	69.17	52.41	40.75
ASR (annual mean per million)	70.03 (58.90–82.65)	52.68 (48.24–56.27)	41.24 (36.79–47.07)
Cases geolocated n (%)	122 (87.85)	445 (94.88)	309 (98.4)
No. census sectors	99	404	266
No. census sectors with cases n (%)	66 (66.6)	205 (50.74)	143 (53.75)

ASR: Age standardized incidence rate.

**Table 2:** Results of scan tests for spatial and spatiotemporal clusters of acute childhood leukemia by city, Colombia 2000–2015.

Type of Cluster Analysis (n)	Bucaramanga	Cali	Medellín
Localized spatial clusters	1	2	2
Localized spatio-temporal clusters	1	1	1
Industrial conglomerates	4	26	14
Industrial conglomerates with spatial clusters	1	13	5
Industrial conglomerates with significant spatial clusters in multivariable model	1	8	4

**Table 3:** Multivariable models for spatial clusters around industrial conglomerates in three capital cities, Colombia 2000–2015.

City	Industrial Conglomerate	Crude Coefficient ^1^	Adjusted Coefficient ^2^	95% CI	*p*-Value
Bucaramanga	Bucaramanga 1	−0.509	−0.220	−0.367–−0.070	0.003
Cali	Cali 17	−0.093	−0.047	−0.096–−0.002	0.059
	Cali 18	−0.106	−0.066	−0.118–−0.015	0.011
	Cali 19	−0.091	–0.056	−0.097–−0.016	0.006
	Cali 20	–0.090	–0.069	−0.113–−0.026	0.002
	Cali 23	–0.066	–0.045	−0.076–−0.0136	0.005
	Cali 24	–0.058	–0.047	−0.076–−0.018	0.001
	Cali 25	–0.063	–0.046	−0.076–−0.015	0.004
	Cali 26	–0.057	–0.045	−0.075–−0.015	0.003
Medellín	Medellín 7	–0.038	–0.657	−0.892–−0.421	0.000
	Medellín 8	–0.188	–0.667	−0.823–−0.512	0.000
	Medellín 10	–0.032	–0.625	−0.921–−0.329	0.000
	Medellín 11	0.015	–0.436	−0.756–−0.116	0.008

^1^ Distance coefficient (per 1 km) for acute childhood leukemia cumulative incidence rate per million. ^2^ Distance coefficient (per 1 km) adjusted by direction (latitude and longitude) and predominant socioeconomic status.

## Supplementary Materials

The following are available online at https://www.mdpi.com/1660-4601/17/21/7925/s1, Table S1. Results of scan tests for spatial and spatiotemporal clusters of acute childhood leukemia in proximity to industrial sources of air pollution in Bucaramanga (*n* = 122 cases); Table S2. Results of scan tests for spatial and spatiotemporal clusters of acute childhood leukemia in proximity to industrial sources of air pollution for Medellin (*n* = 309 cases); Table S3. Results of scan tests for spatial and spatiotemporal clusters of acute childhood leukemia in proximity to industrial sources of air pollution for Cali (*n* = 445 cases).
