# Supplementary material for: Childhood Leukemia in Small Geographical Areas and Proximity to Industrial Sources of Air Pollutants in Three Colombian Cities
Source: Int J Environ Res Public Health. 2020 Oct 29;17(21):7925. doi: 10.3390/ijerph17217925 (PMC7662935; doi:10.3390/ijerph17217925)
Supplement: Supplementary file 1 [file ijerph-17-07925-s001.pdf]

Childhood leukemia in small geographical areas and proximity to industrial sources of air pollutants in three Colombian cities

Supplementary material

S1. Results of scan tests for spatial and spatiotemporal clusters of acute childhood leukemia in proximity to industrial sources of air pollution in Bucaramanga (n= 122 cases)

Cluster	Ratio (Km)	No. sectors included	P value	Relative Risk	Time
Spatial localized clusters					
1	0.81	5	0.001	2.88	
Spatio-temporal localized clusters					
1	0.1	1	0.001	34.43	2010-2011
Spatial clusters around industrial conglomerates					
Bucaramanga 1	0.4	2	0.051	3.85	

S2. Results of scan tests for spatial and spatiotemporal clusters of acute childhood leukemia in proximity to industrial sources of air pollution for Medellin (n=309 cases)

Cluster	Ratio (Km)	No. sectors included	P value	Relative Risk	Time
Spatial localized clusters					
1	3.67	89	0.001	1.87	
2	0.38	2	0.003	11.11	
Spatio-temporal localized clusters					
1	3.49	40	0.002	3.40	2002-2005
Spatial clusters around industrial conglomerates					
Medellín 7	2.64	50	<0.001	1.86	
Medellín 8	3.2	80	<0.001	1.66	
Medellín 10	2.05	25	0.037	1.77	
Medellín 11	3.98	98	<0.001	1.60	
Medellín 13	6.3	106	<0.001	1.59	

*Km

S3. Results of scan tests for spatial and spatiotemporal clusters of acute childhood leukemia in proximity to industrial sources of air pollution for Cali (n=445 cases)

Cluster	Ratio (Km)	No. sectors included	P value	Relative Risk	Time
Spatial localized clusters					
1	7.27	21	<0.001	5.17	
2	0.96	12	0.001	15.82	
Spatio-temporal localized clusters					
1	1.42	35	<0.001	6.42	2003-2006
Spatial clusters around industrial conglomerates					
Cali 5	0.91	8	<0.039	2.61	
Cali 6	1.73	32	<0.038	1.91	
Cali 9	1.13	20	<0.014	3.40	
Cali 12	1.32	18	<0.016	2.44	
Cali 14	3.45	139	<0.001	1.65	

Cali 17	3.07	102	<0.035	1.51	
Cali 18	3.21	129	<0.002	1.53	
Cali 19	1.61	39	<0.001	2.95	
Cali 20	2.37	66	<0.001	1.56	
Cali 23	3.92	107	<0.001	1.75	
Cali 24	5.79	123	<0.001	1.63	
Cali 25	5.62	105	<0.001	1.65	
Cali 26	7.20	129	<0.001	1.71	
